# Sequence variation in human succinate dehydrogenase genes: evidence for long-term balancing selection on *SDHA*

**DOI:** 10.1186/1741-7007-5-12

**Published:** 2007-03-21

**Authors:** Bora E Baysal, Elizabeth C Lawrence, Robert E Ferrell

**Affiliations:** 1 Department of Obstetrics, Gynecology and Reproductive Sciences, University of Pittsburgh School of Medicine, Pittsburgh, PA 15213, USA; 2 Department of Human Genetics, Graduate School of Public Health, University of Pittsburgh, Pittsburgh, PA 15213, USA

## Abstract

**Background:**

Balancing selection operating for long evolutionary periods at a locus is characterized by the maintenance of distinct alleles because of a heterozygote or rare-allele advantage. The loci under balancing selection are distinguished by their unusually high polymorphism levels. In this report, we provide statistical and comparative genetic evidence suggesting that the *SDHA *gene is under long-term balancing selection. *SDHA *encodes the major catalytical subunit (flavoprotein, Fp) of the succinate dehydrogenase enzyme complex (SDH; mitochondrial complex II). The inhibition of Fp by homozygous *SDHA *mutations or by 3-nitropropionic acid poisoning causes central nervous system pathologies. In contrast, heterozygous mutations in *SDHB*, *SDHC*, and *SDHD*, the other SDH subunit genes, cause hereditary paraganglioma (PGL) tumors, which show constitutive activation of pathways induced by oxygen deprivation (hypoxia).

**Results:**

We sequenced the four SDH subunit genes (10.8 kb) in 24 African American and 24 European American samples. We also sequenced the *SDHA *gene (2.8 kb) in 18 chimpanzees. Increased nucleotide diversity distinguished the human *SDHA *gene from its chimpanzee ortholog and from the PGL genes. Sequence analysis uncovered two common *SDHA *missense variants and refuted the previous suggestions that these variants originate from different genetic loci. Two highly dissimilar *SDHA *haplotype clusters were present in intermediate frequencies in both racial groups. The *SDHA *variation pattern showed statistically significant deviations from neutrality by the Tajima, Fu and Li, Hudson-Kreitman-Aguadé, and Depaulis haplotype number tests. Empirically, the elevated values of the nucleotide diversity (% π = 0.231) and the Tajima statistics (*D *= 1.954) in the *SDHA *gene were comparable with the most outstanding cases for balancing selection in the African American population.

**Conclusion:**

The *SDHA *gene has a strong signature of balancing selection. The *SDHA *variants that have increased in frequency during human evolution might, by influencing the regulation of cellular oxygen homeostasis, confer protection against certain environmental toxins or pathogens that are prevalent in Africa.

## Background

Succinate dehydrogenase (SDH; mitochondrial complex II) is an essential enzyme complex that has dual roles in the Krebs cycle and the electron transport chain (ETC) in mitochondria [1]. SDH is composed of four subunits encoded by the nuclear genes *SDHA*, *SDHB*, *SDHC*, and *SDHD*. *SDHA *at chromosome band 5p15 and *SDHB *at chromosome band 1p35 encode the two catalytical hydrophilic subunits flavoprotein (Fp; 70 kDa) and iron-sulfur (Ip; 35 kDa), respectively. *SDHC *at chromosome band 1q23 and *SDHD *at chromosome 11q23 encode the two membrane-spanning hydrophobic subunits, cybL (15 kDa) and cybS (12 kDa), respectively. The *SDHA, SDHB, SDHC*, and *SDHD *gene products are encoded by 15, 8, 6, and 4 exons, which span genomic distances of ~38 kb, 35 kb, 50 kb. and 10 kb, respectively [2,3].

The identification of the *SDHD *subunit gene as the hereditary paraganglioma type 1 locus (PGL1) has uncovered unexpected links between SDH and tumor susceptibility, and highlighted the role of mitochondria in cancer [4]. Since then, mutations in *SDHB*, *SDHC*, and *SDHD *subunit genes (PGL genes) have been established as an important cause of sporadic and familial paragangliomas [5-10]. The paraganglia specificity of PGL tumors [4] and data from global gene-expression analysis [11], cell biology [12], animal-model studies [13], and gene-environment interaction and population genetics [14] support the hypothesis that constitutive hypoxic stimulation underlies the pathogenesis of PGL.

The role of SDH in disease pathogenesis has been implicated independently through a series of studies on a widely distributed plant and fungal neurotoxin, 3-nitropropionic acid (3-NPA)[15]. Acute food poisoning with 3-NPA, which can lead to central nervous system defects with lifelong disability and to mortality in ~10% of the cases, have been associated with consumption of moldy sugarcanes in China [16]. The neurodegeneration induced by 3-NPA poisoning often involves the basal ganglia, hippocampus, spinal tracts, and peripheral nerves, and the symptoms mimic those of Huntington's disease [17]. 3-NPA irreversibly inhibits SDH, owing to the similarity of the chemical structures of 3-NPA to succinate [18]. It has been suggested that 3-NPA may form a covalent adduct with an arginine residue at amino acid position 345 in the active site of the Fp subunit [19].

Surprisingly, mutations in the major catalytical subunit *SDHA *have yet to be associated with PGL. Although homozygous mutations in *SDHA *have been found in Leigh syndrome [20], a severe neurodegenerative disorder of childhood, and with neuromusculopathies, no genetic link between *SDHA *and paraganglioma susceptibility has ever been established. Current biochemical knowledge on SDH provides very few clues for the phenotypic dichotomy arising from the germline subunit gene mutations. *SDHA *and *SDHB *subunits encode the two physically-interacting catalytical subunits, so it is surprising that their mutations would have such different phenotypic consequences [21]. Recently, after identifying cDNA sequences encoding a missense Fp variant containing the Y629F and V657I polymorphisms, Tomitsuka et al [22,23] proposed that distinct genetic loci encode two Fp variants, namely type I and type II. They reached this conclusion after observing tissue-specific and cell line-specific differential expression of the cDNA variants and PCR amplification from genomic DNA of processed *SDHA *gene fragments that lacked introns (i.e, a functional *SDHA *retrogene). However, the genomic location of the retrogene that was proposed to encode the second *SDHA *gene could not be determined. A retrogene for *SDHA *is not present in the human genome, according to the March 2006 assembly in The UCSC database. [24] Finally, Briere et al [25] showed the presence of the missense *SDHA *variants in several different cell types and assumed that these variants originate from two different genes, although they provided no experimental or bioinformatic evidence for the genomic presence of a second *SDHA *locus. Briere et al [25] suggested that the presence of two *SDHA *genes in paraganglia prevents tumorigenesis. If Fp were encoded by two different loci, this would indeed have provided a simple explanation for why *SDHA *mutations would not be associated with PGL susceptibility.

An alternative approach to gain insights into gene function involves analysis of sequence variation in the population. To date, no study has systematically addressed the variation patterns in the SDH subunit genes in normal subjects from different racial or ethnic groups. To gain further insights into the multiple roles of SDH in disease predisposition and to help to integrate the seemingly disparate phenotypic consequences of SDH subunit defects, we examined sequence variation in the complete coding and partial flanking intronic sequences of the four SDH subunit genes in 24 samples from an African American population and 24 samples from a white population. These analyses uncovered an unexpected degree of nucleotide diversity in the *SDHA *gene.

## Results

### Sequence variants in the SDH subunit genes

**Figure 1:**
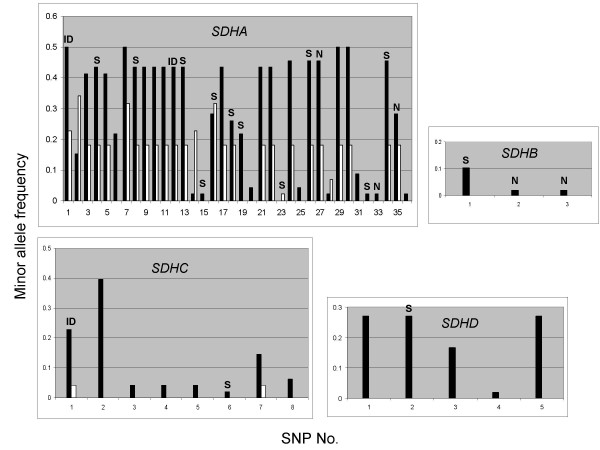
**Minor allele frequency of each SDH subunit variant (also see Additional Table 1) is shown**. Filled vertical bars refer to African American, unfilled vertical bars refer to European American samples. Synonymous and non-synonymous coding variants are marked by S and NS, respectively. ID refers to insertion/deletion polymorphisms.

Using PCR, each coding exon and the flanking introns of the four SDH subunit genes in 24 European American and 24 African American samples were amplified, and were then sequenced. In total, 3828 coding and 7013 non-coding nucleotides were sequenced for each sample, and 52 polymorphisms were detected (Table 1). The heterozygous frequencies of all variants were consistent with Hardy-Weinberg expectations (p > 0.01) in both sets of samples. Except for two non-coding indels in *SDHA *and one in *SDHC*, all variants were single nucleotide polymorphisms (SNPs) involving base replacements. A full list of the identified sequence variants is provided in Additional File 1 and has also been submitted to the SDH mutation database [26]. *SDHA *variant density was 2.6-fold and 2.3-fold higher in the coding and non-coding regions, respectively, than the average of 106 genes [27]. The minor allele frequencies of all variants are shown in Figure 1.

### Nucleotide diversity in SDH subunit genes

We calculated the nucleotide diversity in SDH subunit genes using the population genetic parameters π and θ_s _(Table 2). As expected, all diversity indices were higher in the African American samples. The nucleotide diversity (%) in the total sample set was low at the PGL genes: *SDHB *(π = 0.008), *SDHC *(π = 0.065), and *SDHD *(π = 0.044). In contrast, the nucleotide diversity of the *SDHA *gene (π = 0.199%) was 5.1-fold higher than the average of the PGL genes and 3.4-fold higher than the average (π = 0.058%) of 292 autosomal genes [28]. The θ_s _and π estimates of nucleotide diversities were similar for the membrane-spanning subunits *SDHC *and *SDHD*, but differed substantially for the two catalytic subunits. Whereas the π estimate was ~1.6-fold higher than θ_s _for the *SDHA *gene, consistent with the enrichment of alleles with intermediate frequencies, the θ_s _estimate was ~4-fold higher for the *SDHB *gene, indicating the very low frequency of the allelic variants. For comparison, 90% of the genes in a recent survey had θ_s _estimates higher than the π estimates [28], indicating an abundance of rare alleles, which is thought to be a result of recent population expansion in humans. F_ST _statistics provided statistically significant evidence of population differentiation between the two racial groups for the *SDHA*, *SDHC*, and *SDHD *genes, but not for the *SDHB *gene (Table 2). This was attributable to the very low frequencies and the absence of *SDHB *allelic variants in the African American and European American samples, respectively.

### Comparison of the human and chimpanzee *SDHA *genes for sequence diversity

To test whether high nucleotide diversity also characterizes the chimpanzee *SDHA *gene, we used the human PCR primers to amplify and sequence 18 unrelated chimpanzee samples. We obtained high-quality sequences for exons 3–6, 8, 12, 13, and 15, which together comprise a total genomic sequence size of 2832 bp (Table 1). We identified one silent exonic and seven intronic fixed-nucleotide differences between the human and chimpanzee *SDHA *genes (Additional file 2), corresponding to a substitution rate of 0.28%. The nucleotide substitution rate in *SDHA *is lower than the average of 127 known genes (0.75%) that were recently sequenced in human and chimpanzee [29]. The chimpanzee *SDHA *gene has 10 polymorphic variants, compared with 21 in the human gene in the same region, and showed ~2.9-fold lower nucleotide diversity (π) than the human gene (Table 3). Furthermore, θ_s _and π estimates of nucleotide diversities were similar in the chimpanzee, consistent with neutral expectations. These findings indicate that the mutation rate in *SDHA *is not inherently high and that the increased nucleotide diversity in the human gene must have occurred after the split of the two species from their common ancestor 5–6 million years ago.

### Tests of neutrality

We employed three commonly used tests (the Tajima *D *tests and the Fu and Li D* and F* tests) to identify departures of the allelic distributions from neutral expectations. None of the PGL genes showed statistically significant departures from neutrality in samples from either racial group (Table 4). In contrast, the allelic distribution of the *SDHA *gene showed positive test values at statistically significant levels in both racial samples (Table 5). Notably, the neutrality statistics were supportive of balancing selection on *SDHA *despite the presence of six singleton variants in the African American samples and one singleton variant in the European American samples (Additional file 1). To obtain a clearer picture of the departure of *SDHA *allelic distributions from neutral expectations, we analyzed non-coding, coding, synonymous, and non-synonymous variants separately (Table 5). Nominally significant departures from neutrality were obtained in seven of the nine test statistics for the non-coding variants, although the *SDHA *coding region variation was also suggestive of an excess of variants in intermediate frequencies in the African American samples.

To test whether the level of silent diversity in *SDHA *correlates with level of divergence between human and chimpanzee, as predicted by the neutral theory, we used the Hudson-Kreitman-Aguadé (HKA) test. Sequence data from four loci that were assumed to be evolving neutrally were used for comparison. These loci include non-coding regions on chromosome bands 1q24 [30], 22q11 [31], and Xq13.3 [32] and the promoter region of β-globin at 11p15 [33]. Locus-by-locus comparison provided statistical significance in two of the four tests, suggesting increased diversity in *SDHA *relative to these two loci (Additional file 3). To further address whether the *SDHA *variation pattern is unusual when information from the comparison loci is jointly used, we used a recently developed maximum-likelihood-ratio test [34]. The likelihood of two models were compared; the first assumes that all five loci evolve neutrally, whereas the other assumes that *SDHA *is subject to selection while the other four loci evolve neutrally. The model assuming selection on *SDHA *was statistically supported over the model of neutrality (*p *= 5.3 × 10^-3^; Table 6). These results further support the hypothesis that increased nucleotide diversity in *SDHA *is maintained by balancing selection.

### Empirical assessment of neutrality in SDH subunit genes

**Figure 2:**
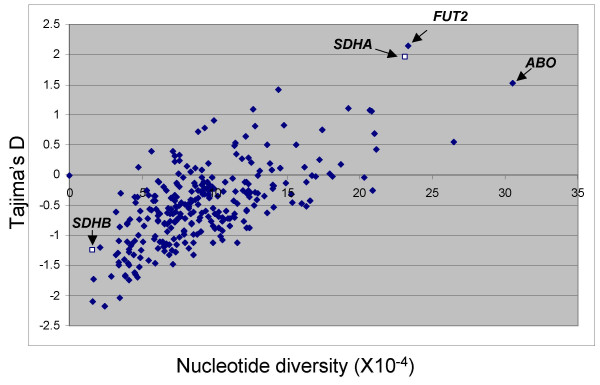
**Tajima's *D *statistic and nucleotide diversity in *SDHA *and *SDHB *genes (squares) relative to the 282 genes in SeattleSNP database are shown in the African American population**. Positions of two highly polymorphic loci, *ABO *and *FUT2*, the latter of which encodes the alpha(1,2)fucosyltransferase, are shown.

Because population history plays an important role in shaping the variation patterns in the genome, we sought to assess whether the nucleotide diversity of complex II genes were unusual compared with other genes across the genome. We used the summary statistics for Tajima's *D *test and nucleotide diversity of 282 genes listed in the SeattleSNP database for comparison. When compared with the database genes, the statistics for nucleotide diversity and Tajima's *D *were not outstanding for any of the complex II genes in the European American samples or for the *SDHC *and *SDHD *genes in the African American samples. However, the *SDHA *nucleotide diversity was higher than that of 279 (*p *< 0.015) of the genes and the Tajima *D *statistic was higher than that of 281 (*p *< 0.0036) of the genes in the African American samples (Figure 2). In contrast, *SDHB *had less sequence diversity than 280 of the SeattleSNP genes (*p *< 0.011) in the African American samples. A recent analysis of 151 loci in the SeattleSNP set has indicated that the *D *statistic of the *ABO *locus (*D *= 1.58) retains its significance in an African American population under several demographic scenarios [35]. Because the magnitude of *D *in *SDHA *in our African American samples (*D *= 1.95) is higher than that in the *ABO *locus (Figure 2), it is likely that the statistical support for balancing selection on *SDHA *would be retained by different population histories. In summary, the departure of *SDHA *allelic distribution from neutral expectations is empirically supported in the African American samples, consistent with a balancing selection mechanism.

### Haplotype structures of the SDH subunit genes

Haplotypes, haplotype-block structures and the tagging SNPs for each block were inferred using the web-based HAP software (see methods). As expected, the haplotypes were more variable in the African American than in the European American samples. The *SDHA *haplotype variation could be defined by 6 haplotype blocks and 13 tagging SNPs in the African American samples but only by 3 haplotype-blocks and 5 tagging SNPs in the European American samples (Additional file 1). In contrast, haplotype variation in the PGL genes could be defined by single-haplotype blocks. The most common haplotype accounted for ~99% of the haplotypes of the PGL genes in the European American samples (Additional file 4). Similarly, the most common haplotype and its 1-nucleotide neighbors covered ~98%, 79% and 73% of the variation in the *SDHB*, *SDHC*, and *SDHD *genes, respectively, in the African American samples.

**Figure 3:**
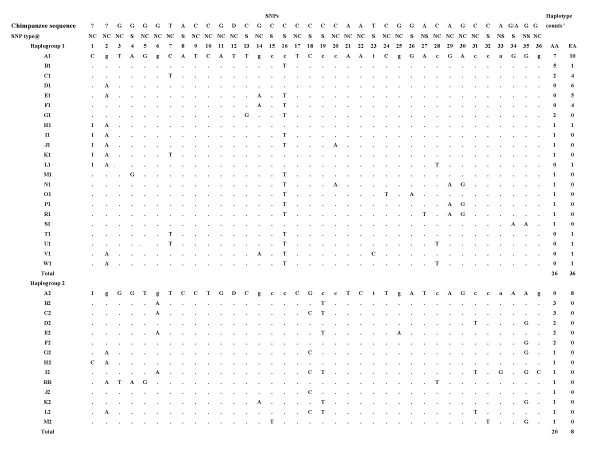
**Haplotypes of *SDHA***. Periods denote the identical SNP variant when compared to the most common haplotype of each haplogroup. @NC = non-coding, S = synonymous coding, NS = non-synonymous coding. I = insertion allele, D = deletion allele. ^AA = African-American samples, EA = European-American samples.

The commonness of a single haplotype and its 1-nucleotide neighbors in the PGL genes was in stark contrast to the presence of two common but highly dissimilar haplotypes in *SDHA *in both racial groups. The two most common *SDHA *haplotypes, A1 and A2, accounted for ~19% (17/90) and ~9% (8/90) of all haplotype diversity, respectively, and differed from each other in 22 of the 36 variant positions (Figure 3). Haplotype A1 and A2 encode the missense Fp variants Y629-V657 and F629-657, by the SNPs 27 and 35, respectively, indicating an allelic association of the missense variants in these two amino-acid sites. Notably, the variant Fp amino acids Y629 and V657 were conserved in mammalian Fp sequences, including orangutan, macaque, mouse, dog, rat, and bovine. However, different amino acids were found in phylogenetically more distant species such as the zebrafish, which had Y629-I657 and *Dirofilaria*, an infectious nematode, which had E629-I657.

**Figure 4:**
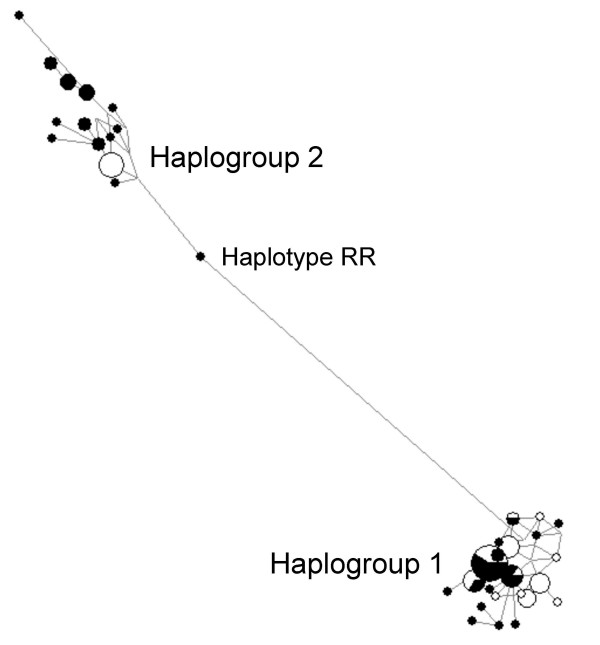
**A median-joining network groups all *SDHA *haplotypes (Figure 3) on the basis of number of nucleotide differences**. The haplotype RR is probably a recombinant between the two haplogroups. The pie chart for each haplotype depicts the proportional contribution of the African American (filled portion) and the European American (unfilled portion) samples.

All of the remaining 34 *SDHA *haplotypes were highly similar to one of the two commonest haplotypes, and formed two distinct haplotype sets, referred to as haplogroup 1 and haplogroup 2. The haplotypes within each group differed from the most common haplotype of the group in up to seven variant positions, with a median number of three differences. The frequencies of haplogroups 1 and 2 were ~56% and ~44% in the African American samples and ~82% and ~18% in the European American samples, respectively. A median-joining network of all haplotypes clustered all but one haplotype within two distinct haplogroup clusters (Figure 4). The only haplotype (RR) that mapped outside of the two haplogroups clusters was probably a recombination product between haplogroup 1 and haplogroup 2.

### Haplotype number test

To test whether the number of predicted *SDHA *haplotypes in the African American samples is compatible with neutral evolution, we employed the Depaulis and Veuille haplotype number test [36]. In total, 35 variants in 46 African American sequences defined 27 different haplotypes (Figure 3). Using Depaulis and Veuille simulations under assumptions of neutrality showed that when there are 40 variants in 50 sequences, the upper limit of the 95% confidence interval for the expected number of different haplotypes is 24. Thus, the number of *SDHA *haplotypes is statistically significantly higher than expected under neutrality, and is consistent with an ancient balanced polymorphism in the African American population.

### Estimating age of the *SDHA *haplogroups

We estimated the age of the two haplogroups by comparing the sequence divergence between them with that between the human and chimpanzee genes, assuming a constant evolutionary rate of nucleotide substitutions. Haplogroups 1 and 2 have eight fixed nucleotide differences, at SNPs 8–12, 17, 21, and 22 (Figure 3), within 5255 bp, whereas human and chimpanzee genes have eight fixed nucleotide differences within 2832 bps. On the basis of these fixed nucleotide substitutions, we estimated haplogroups 1 and 2 to be as old as [(8/5255)/(8/2832)] times the divergence time of human and chimpanzees. Thus, *SDHA *balanced polymorphisms were estimated to be 2.69–3.23 million years old, assuming a divergence time of 5–6 million years for human and chimpanzees. This is probably a conservative estimate, as the fixed differences between the haplogroups erode in time by recombination and gene conversion.

## Discussion

Our results establish a foundation to understand the selective and demographic forces that have shaped the variation patterns in SDH subunit genes, and have important functional implications. Our findings indicate that the variation pattern in *SDHA *is characterized by the presence of higher sequence diversity, two common and highly dissimilar haplogroups, and statistical and empirical support for the operation of a balancing selection mechanism. Our data also refute the previous suggestions that the Y629F and V657I variants originate from two distinct genetic loci because these missense variants are encoded by a single, highly polymorphic *SDHA *gene.

The PGL genes had much lower nucleotide diversity, which was especially evident in *SDHB*, suggesting that the *SDHB *gene product might be under functional constraints that preclude the accumulation of variants. If slightly deleterious variants in PGL genes increase the risk of paraganglioma tumor development, such variants would be eliminated before they reach high frequencies in the population. This potential mechanism might apply especially to *SDHB *because its mutations are associated with malignancy and early-onset pheochromocytomas that could lead to severe hypertensive crises [37,38]. In contrast, because there is no evidence that heterozygous mutations in *SDHA *are associated with a pathologic phenotype, negative selection of deleterious *SDHA *alleles may operate only when they are in the homozygous state, which often leads to a lethal metabolic syndrome in childhood.

A major finding of our study is the unexpectedly high nucleotide diversity in the *SDHA *gene in the African American samples. It has been suggested that high local recombination rates may increase SNP density [39]. However, this mechanism is unlikely to contribute to *SDHA *variant density, because a recent high-resolution recombination map indicates a very low recombination rate at the tip of chromosome 5 short arm, where *SDHA *is located [40]. It is conceivable that the four *SDHA *pseudogenes, generated by complete or partial gene duplications, may increase the *de novo *mutation rate in the *SDHA *gene through illegitimate recombination or gene conversion during meiosis to increase variant density. However, lack of high nucleotide diversity in the chimpanzee *SDHA *gene does not suggest that the mutation rate in *SDHA *is inherently high, even though the chimpanzee genome also contains the duplicated *SDHA *pseudogenes. Rather, our findings suggest that the high nucleotide diversity of the human *SDHA *gene is a consequence of persistence of two distinct haplogroups for long periods during human evolution, leading to acquisition of a distinct set of polymorphisms by each haplogroup.

The most important finding of our study is the statistical and empirical support for a balancing selection mechanism on *SDHA*. A classic example of balancing selection is found at the major histocompatibility complex (MHC) loci [41], where high levels of polymorphisms in the functional MHC genes may confer a selective advantage to the heterozygotes by enabling them to process a wider range of pathogen antigens on T cells. The variation in a few other human genes may also have been shaped by balancing selection. For example, the 5' *cis*-regulatory region of *CCR5*, encoding the principal coreceptor for HIV-1 [42], protocadherin alpha gene cluster promoters [43] and the bitter-taste receptor gene, *PTC *[44], have two major ancient haplotype groups and positive *D *test statistics, similar to *SDHA*. However, in contrast to *SDHA*, these genes did not show significant Tajima *D *statistics in the African or African American samples. In general, the average Tajima *D *value is positive in the European American population and negative in the African American population. Positive Tajima *D *statistics in European Americans are often interpreted to reflect population contraction that occurred during the migration of modern humans out of Africa, whereas negative Tajima *D *statistics in African Americans may reflect admixture between African and European populations [35]. Thus, evidence of balancing selection on a gene, suggested by statistically significantly positive Tajima *D *values, is more likely to be confounded by population history in European American samples than in African American samples.

It is conceivable that an environmental factor prevalent in Africa may have contributed to the increased frequency of certain *SDHA *variants that might have differential roles in the regulation of oxygen homeostasis by the SDH complex. A candidate environmental factor is the neurotoxin 3-NPA and its aliphatic nitrocompounds derivatives. In addition to being a product of certain fungi such as *Arthrinium *species, 3-NPA and its derivatives are also found in several higher plants. The toxicity of these plants is well established, because their aliphatic nitrocompound contents have been linked to acute and chronic diseases in some domestic animals. Major livestock losses were attributed to plant nitrocompounds in the western United States, Canada and Mexico [45]. Thus, although human toxicity involving moldy sugarcane poisoning have to date been reported only in China, human exposure to 3-NPA and other nitrocompounds might be more common throughout the world than is indicated by the numbe of clinical cases [18,46]. 3-NPA exposure might be more prevalent in Africa partly because a hot and humid climate promotes the growth of fungi. If certain *SDHA *variants confer a selective advantage against 3-NPA poisoning by affecting gene expression levels, protein translation efficiency, and/or the binding affinity for 3-NPA, then such variants may provide a survival advantage for their carriers against 3-NPA poisoning. Alternatively, SDH may play a currently unrecognized role against infectious pathogens such as malaria, which are prevalent in Africa. Genetic studies of PGL suggest that inactivation of SDH by subunit mutations inappropriately activates hypoxia-inducible pathways. If the *SDHA *variants that have increased in frequency during human evolution are hypomorphs or encode Fps that have slight functional deficits, these variants might promote the activation of hypoxia-inducible pathways and help the immune cells to survive better under sustained hypoxic microenvironments of the infected tissues.

Finally, our findings do not support the previous explanations as to why *SDHA *mutations are not associated with PGL susceptibility because these explanations assume the presence of two *SDHA *genes in the human genome. Instead, the contrasting patterns of sequence variation between *SDHA *and the PGL genes suggest the presence of two functionally distinct modules in SDH: one formed by the three closely-associated PGL gene products (PGL module), and the other a loosely-interacting, highly-variable *SDHA *protein product. This model provides an alternative explanation as to why *SDHA *mutations do not cause PGL and predicts the following two conditions:

(i) The relative concentration of *SDHA *protein product is much higher (>two-fold) than the PGL module in the paraganglionic tissues. Thus, even a 50% reduction in *SDHA *protein levels, as a result of heterozygous mutations, would not compromise the SDH function in paraganglia to initiate tumor formation.

(ii) The physical interaction between the *SDHA *protein product and the PGL module is loose and kinetically fast during catalysis, thus a mutant *SDHA *protein product could not irreversibly trap a PGL module to initiate tumor formation.

## Conclusion

Our findings demonstrate that the *SDHA *gene carries a strong signature of balancing selection in the African American population and that PGL and *SDHA *gene products are subject to distinct selective constraints. Collectively, these data provide new insights into SDH biology and may catalyze further research on the causes and the consequences of the unexpectedly high sequence diversity in the *SDHA *subunit gene.

## Methods

### Samples

DNA was isolated using standard protocols from samples from 24 unrelated African American and 24 unrelated European American women, which are part of an anonymized sample collection in the Department of Human Genetics at The University of Pittsburgh School of Public Health. The samples were collected under research protocols approved by the internal review board review committee. One African American and two European American samples that failed to amplify multiple *SDHA *exons on repeated attempts were removed from certain analyses, including minor allele frequency calculations, haplotype analysis, and neutrality statistics. We also sequenced the *SDHA *gene in 18 unrelated common chimpanzees (*Pan troglodytes*), which are part of the primate DNA collection in the Department of Human Genetics.

### PCR and sequencing

PCR amplification for each exon was performed by using oligonucleotide primers that were designed from the flanking intronic or untranslated sequences of the exons. The primer sequences and the amplicon sizes for each SDH subunit gene exon are provided in Additional file 5. The PCR amplification was performed using *Taq *polymerase under standard conditions. The PCR amplification of *SDHA *is potentially confounded by the presence of multiple pseudogenes created by genomic duplications. These pseudogenes contain multiple mutations in their coding regions. BLAST analyses of human expressed sequences database in GenBank reveal no evidence for expression of the *SDHA *pseudogenes (data not shown). The PCR primers for specific amplification of the *SDHA *gene were designed so that the 3' ends of the primers were placed at nucleotides that showed divergence from the pseudogenes. The human genome March 2006 sequence assembly at UCSC database indicates that *SDHA *has two complete and one truncated gene duplications within ~3 Mb at chromosome band 3q29 and one truncated duplication ~100 kb centromeric to the functional gene at chromosome band 5p15 [24]. The duplicated *SDHA *copies have 92.5–98.4% sequence identity with the functional gene within the exons and in the flanking introns. This high degree of sequence identity has erroneously led to the designation of some of the fixed nucleotide differences between the functional *SDHA *gene and its pseudogenes as real SNPs in the *SDHA *gene in the dbSNP database. In our experiments, we confirmed the specific amplification of each *SDHA *exon by analyzing the nucleotide positions of the amplicons where there are fixed differences between the functional and the duplicated gene copies (number of fixed nucleotide differences between *SDHA *and its duplicated pseudogenes are indicated in Additional file 5). In addition, we confirmed that all *SDHA *exonic variants, except the rare variants of SNPs 15, 33, and 36, which were observed only once in our whole sample set (i.e. were singletons), are represented by multiple expressed sequence tags (ESTs) in the human EST database at NCBI as determined by BLAST analyses [47]. Taken together, these results confirm that our genomic primers have specifically amplified the exons of the functional *SDHA *gene while avoiding the duplicated pseudogenes.

### Computational analyses

The sequenced segments of the genes, including the coding, non-coding and flanking intronic sequences, were conjoined in a single gene-sequence file. This file was then used to enter polymorphism data for each sample using Sequencher™ software (Gene Codes Corporation, Ann Arbor, MI, USA). The sequence files for each sample were used to generate input files for data analyses in population genetic software. Nucleotide diversity, population diversification analyses and departures from Hardy-Weinberg expectations were calculated using Arlequin software (version 2.001) [48]. Tests of neutrality were conducted using DnaSp software (version 4.10) [49]. The phylogenic relationship between the inferred haplotypes was established using Network software (version 4.1) [50]. All software programs were operated on a PC platform. Haplotype analyses and the prediction of tagging SNPs were performed using HAP, a free web-based haplotype analysis software.

### Sequence databases

We used the BLAT function of UCSC genome browser to determine the genomic locations of and sequence similarities between *SDHA *genomic duplications [24]. The Ensembl genome browser was used to determine the intron-exon junction, transcription initiation sites, and start/stop codons of the SDH subunit genes [51]. Gene variation data in the SeattleSNP database (August 2006) derived from 24 African American individuals and 23 Europeans [52] were used to compare with our results.

### Population genetics

#### Nucleotide diversity

Two measures of nucleotide diversity were derived using unphased genotypic data: π, which measures the mean number of differences per nucleotide between two randomly chosen sequences and θ_s_, which measures the proportion of segregating sites under the assumption of an infinite site-neutral model. Both measures estimate the mutation rate, θ_s _= 4N_e_μ, where N_e _is the effective population size and μ is the neutral mutation rate per generation.

In a sample of n chromosomes, π = Σ_i<j _π_i, j_/n_c_, where π_i, j _is the number of nucleotide differences between *i*th and *j*th DNA sequences and n_c _= n(n - 1)/2 and

θ_s _= S/a, where a=∑i=1n−11/i
 MathType@MTEF@5@5@+=feaafiart1ev1aaatCvAUfKttLearuWrP9MDH5MBPbIqV92AaeXatLxBI9gBaebbnrfifHhDYfgasaacH8akY=wiFfYdH8Gipec8Eeeu0xXdbba9frFj0=OqFfea0dXdd9vqai=hGuQ8kuc9pgc9s8qqaq=dirpe0xb9q8qiLsFr0=vr0=vr0dc8meaabaqaciaacaGaaeqabaqabeGadaaakeaacqqGHbqycqGH9aqpdaaeWbqaaiabigdaXiabc+caViabbMgaPbWcbaGaeeyAaKMaeyypa0JaeGymaedabaGaeeOBa4MaeyOeI0IaeGymaedaniabggHiLdaaaa@3AFB@

#### Tests of neutrality

θ_s _is strongly affected by the existence of deleterious alleles, because such alleles are usually present in low frequencies, but θ_s _is not affected by the frequency of mutants. Conversely, π is not significantly affected by the presence of rare deleterious alleles because π incorporates the frequency of mutants. If some of the variants in the sample have selective effects, then the estimates of θ_s _and π will be different. Tajima [53] used the difference between these two estimates to detect selection among the sequences.

Tajima's *D *statistic is calculated as *D *= (π - θ_s_)/[Var(π - θ_s_]^1/2^

The value of *D *is expected to be zero for selectively neutral variants in a constant population. A non-zero *D *value is a sign of departure from the neutral model caused by a relative excess (positive *D *values) or deficiency (negative *D *values) of substitutions of various frequencies [54].

Departures from the neutral model of the allelic distributions can also be tested by Fu and Li's D* and F* test statistics [55]. These tests compare the number of mutations between internal and external branches of a sequence genealogy with their expectations under selective neutrality. D* and F* tests compare the number of nucleotide variants observed only once in a sample with the total number of nucleotide variants and with the mean pairwise difference between the sequences, respectively. We assessed the significance of neutrality test statistics by comparing the observed test values to those obtained by 10000 coalescent simulations using sample size and number of segregating sites as variables and assuming a standard neutral model with no recombination. Coalescent simulations were performed by DnaSp software (version 4.10).

We used the HKA test for excesses of variation in *SDHA *gene. This test compares whether the level of intra-specific polymorphism parallels the level of nucleotide divergence between two species in a given locus relative to neutrally evolving loci. We used the direct HKA mode in the DNAsp software for locus-by-locus comparison. We also used a software testing maximum likelihood ratio of selection on *SDHA *in a multilocus framework as described previously [34]. Twice the difference of log likelihoods for two competing models is approximately χ^2 ^distributed, with the degree of freedom (d.f.) equal to the number of selected loci. We seeded 100000 and 200000 cycles of the Markov chain to run two independent tests on a PC. Both chains provided similar results.

#### Genetic structure of populations

The genetic structure of populations was investigated by the analysis of molecular variance (AMOVA) approach, as implemented in Arlequin software [48]. This approach is based on the analyses of variance of gene frequencies. The proportion of total variation among populations is estimated by F_ST_, Wright's fixation index.

### Haplotype analyses

We used HAP, a software employing a highly accurate method for common haplotype prediction from genotype data [56] to calculate minor allele frequencies of all variants. The haplotype resolution employs a phasing method that uses imperfect phylogeny. This method partitions the SNPs into haplotype blocks, and for each block, it predicts the common haplotypes and each individual's haplotype. We used Network (version 4.1), a phylogenetic network analysis software, to generate an evolutionary tree network that links the predicted haplotypes on the basis of their similarity [50].

## Authors' contributions

BEB and REF conceived and designed the study. BEB performed the statistical analyses and drafted the manuscript. REF and ECL revised the manuscript critically for important intellectual content. ECL performed the sequence analyses. BEB and REF obtained funding. All authors read and approved the final manuscript.

## Supplementary Material

Additional file 1 Additional Table 1 – S DH subunit gene variants Click here for file

Additional file 2 Additional Table 2 – Sequence variants and fixed differences in chimpanzee *SDHA* Click here for file

Additional file 3 Additional Table 3 – Locus-by-locus HKA tests of *SDHA *versus neutrally evolving loci Click here for file

Additional file 4 Additional Table 4 – Haplotype structures of *SDHB*, *SDHC*, and *SDHD* Click here for file

Additional file 5 Additional Table 5 – PCR oligonucleotide primers and the amplicon sizes for *SDHA, SDHB, SDHC*, and *SDHD *exons Click here for file

## Figures and Tables

**Table 1:** Summary of variants in SDH subunit genes

Gene	No. of coding base pairs sequenced	No. of synonymous variants	No. of non-synonymous variants	No. of non-coding base pairs sequenced	No. of non-coding variants
*SDHA*	1995	12	3	3 275	21
*SDHB*	843	1	2	879	0
*SDHC*	510	1	0	1303	7
*SDHD*	480	1	0	1556	4
Chimpanzee *SDHA*	1119	3	0	1713	7

**Table 2:** Sequence diversity in succinate dehydrogenase subunit genes

Locus	Length (bp)*	Population	Sample size (*n*)†	S‡	Gene diversity ± SD	Nucleotide diversity, % (θ ± SD)	Nucleotide diversity, % (π ± SD)	F_ST _(P§)
*SDHA*	5270	AA	24	35	0.974 ± 0.010	0.141 ± 0.045	0.231 ± 0.118	0.093 (0.012)
		EA	24	27	0.903 ± 0.030	0.107 ± 0.036	0.147 ± 0.077	
		All	48	36	0.964 ± 0.009	0.126 ± 0.037	0.199 ± 0.101	
*SDHB*	1722	AA	24	3	0.231 ± 0.078	0.039 ± 0.024	0.016 ± 0.019	0.061 (0.113)
		EA	24	0	0	0	0	
		All	48	3	0.120 ± 0.045	0.034 ± 0.021	0.008 ± 0.013	
*SDHC*	1813	AA	24	8	0.680 ± 0.060	0.086 ± 0.040	0.103 ± 0.067	0.176 (~0)
		EA	24	2	0.082 ± 0.053	0.012 ± 0.012	0.013 ± 0.017	
		All	48	8	0.441 ± 0.061	0.075 ± 0.033	0.065 ± 0.046	
*SDHD*	2036	AA	24	5	0.609 ± 0.063	0.055 ± 0.028	0.077 ± 0.052	0.234 (~0)
		EA	24	0	0	0	0	
		All	48	5	0.361 ± 0.059	0.048 ± 0.024	0.044 ± 0.034	

AA, African American samples; EA, European American samples; SD, standard deviation.

*Includes coding and non-coding sequences.

†Number of unrelated subjects.

‡Number of segregating sites.

§*p *value for population genetic structure between AA and EA.

**Table 3:** Sequence diversity in the human and chimpanzee *SDHA *genes

Species	Length (bp)	Sample size (*n*)	S*	Nucleotide diversity, % (θ_s _± SD^c^)	Nucleotide diversity, % (π ± SD)
Human	2832				
AA		23	20	0.153 ± 0.054	0.279 ± 0.147
EA		22	16	0.122 ± 0.046	0.168 ± 0.093
All		45	21	0.139 ± 0.045	0.238 ± 0.125
Chimpanzee	2832	18	10	0.077 ± 0.033	0.082 ± 0.051

AA, African American samples; EA, European American samples; SD, standard deviation.

*Number of segregating sites.

**Table 4:** Tests of Neutrality in PGL genes

Locus	Length (bp)*	Population	Sample size (*n*)†	S‡	% θ_s_	% π	Tajima *D *test statistic	Fu and Li D* test statistic	Fu and Li F* test statistic
*SDHB*	1710	AA	24	3	0.04	0.016	-1.24 (0.117)	-1.70 (0.152)	-1.82 (0.080)
	1710	EA	24	0	0	0	--	--	--
	1710	All	48	3	0.034	0.008	-1.37 (0.071)	-1.98 (0.113)	-**2.10 (0.044)**
*SDHC*	1806	AA	24	7	0.087	0.064	-0.724 (0.266)	0.472 (0.186)	0.107 (0.463)
	1806	EA	24	1	0.012	0.005	-0.87 (0.362)	0.55 (0.237)	0.16 (0.238)
	1806	All	48	7	0.075	0.039	-1.150 (0.126)	0.328 (0.233)	-0.201 (0.411)
*SDHD*	2033	AA	24	5	0.055	0.076	0.887 (0.166)	0.144 (0.282)	0.438 (0.329)
	2033	EA	24	0	0	0	--	--	--
	2033	All	48	5	0.048	0.044	-0.195 (0.490)	-0.017 (0.334)	-0.089 (0.461)

AA, African American samples; EA, European American samples; SD, standard deviation.

Significant *p *values (within parentheses) are in **bold**.

*Includes coding and non-coding sequences.

†Number of unrelated subjects.

‡Number of segregating sites. DnaSp software excludes from the analyses the insertion/deletion variants, one of which is in *SDHC*.

**Table 5:** Tests of neutrality in *SDHA*

Locus	Length (bp)	Population	Sample size (*n*)*	S†	% θ_s_	% π	Tajima *D *test statistic	Fu and Li D* test statistic	Fu and Li F* test statistic
Coding	1995	AA	23	14	0.16	0.238	1.515 (0.053)	-0.352 (0.428)	0.320 (0.367)
	1995	EA	22	10	0.115	0.147	0.797 (0.181)	0.805 (0.098)	0.945 (0.154)
	1995	All	45	15	0.148	0.204	1.046 (0.116)	-1.065 (0.210)	-0.361 (0.361)
Synonymous	1995	AA	23	11	0.125	0.191	1.542 (0.051)	-0.249 (0.491)	0.399 (0.344)
	1995	EA	22	8	0.092	0.116	0.726 (0.192)	0.612 (0.416)	0.761 (0.215)
	1995	All	45	12	0.119	0.163	1.003 (0.132)	-0.988 (0.235)	-0.339 (0.383)
Non-synonymous	1995	AA	23	3	0.034	0.047	0.810 (0.197)	-0.390 (0.449)	-0.036 (0.495)
	1995	EA	22	2	0.023	0.031	0.597 (0.231)	0.761 (0.428)	0.827 (0.236)
	1995	All	45	3	0.03	0.041	0.687 (0.215)	-0.561 (0.499)	-0.199 (0.453)
Non-coding	3275	AA	23	19	0.133	0.217	**2.046 (0.015)**	0.908 (0.084)	**1.534 (0.036)**
	3275	EA	22	15	0.106	0.145	1.176 (0.092)	**1.565 (0.038)**	**1.696 (0.020)**
	3275	All	45	19	0.115	0.195	**2.020 (0.019)**	**1.707 (0.025)**	**2.174 (0.005)**
Coding and non-coding	5255	AA	23	33	0.143	0.225	**1.954 (0.017)**	0.402 (0.279)	1.128 (0.091)
	5255	EA	22	25	0.11	0.17	1.114 (0.106)	**1.452 (0.037)**	**1.584 (0.029)**
	5255	All	45	34	0.128	0.198	**1.721 (0.036)**	0.521 (0.228)	1.183 (0.079)

AA, African American samples; EA, European American samples; SD, standard deviation.

Significant *p *values (within parentheses) are in **bold**.

*Number of unrelated subjects.

†Number of segregating sites. DnaSp software excludes from the analyses the insertion/deletion variants, two of which were detected in the non-coding *SDHA *sequence. Inclusion of the *SDHA *indels, by recoding them as SNPs, increased all *SDHA *test statistics and reduced the *p *values (data not shown)

**Table 6:** Maximum-likelihood HKA analysis of silent polymorphisms in *SDHA *relative to four other neutrally evolving loci

Model	Description	Ln likelihood	Likelihood-ratio statistic (d.f.)	*P*	k*				
					
					*SDHA*	1q21	β-globin	22q11	Xq13
1	All neutral	-38.09	--	-	1	1	1	1	1
2	Selection on *SDHA*	-34.2	7.777 (1)	5.3 × 10^-3^	4.7	1	1	1	1

*Relative measure of diversity.
