# Supplementary material for: Sequence variation in human succinate dehydrogenase genes: evidence for long-term balancing selection on SDHA
Source: BMC Biol. 2007 Mar 21;5:12. doi: 10.1186/1741-7007-5-12 (PMC1852088; doi:10.1186/1741-7007-5-12)
Supplement: Additional file 1 — Additional Table 1 – S DH subunit gene variants [file 1741-7007-5-12-S1.doc]

**Additional Table 1 - Sequence variants** in SDH subunit genes

| **Subunit gene** | **SNP No.** | **Sequence** | **Location** | **Coding effect** | **Minor allele (AA/CC)** | **HB tag (AA)** | **HB tag (CC)** |
| --- | --- | --- | --- | --- | --- | --- | --- |
| ***SDHA*** | **1** | **-84 Indel C** | **5'-UTR** | **-** | **=/Ins** | **1*** | **1*** |
|  | **2** | **IVS1 +65 G>A** | **Intron 1** | **-** | **A/A** | **1*** | **1*** |
|  | **3** | **IVS2 -57 T>G** | **Intron 2** | **-** | **G/G** | **1** | **1** |
|  | **4** | **c.309 A>G** | **Exon 3** | **A103A** | **G/G** | **1** | **1** |
|  | **5** | **IVS3 +56 G>T** | **Intron 3** | **-** | **T/T** | **1** | **1** |
|  | **6** | **IVS4 +32 G>T** | **Intron 4** | **-** | **A/-** | **1*** |  |
|  | **7** | **IVS4 -57 C>T** | **Intron 4** | **-** | **C/T** | **2*** | **1*** |
|  | **8** | **c.619 A>C** | **Exon 5** | **R207R** | **C/C** | **2*** | **1** |
|  | **9** | **IVS5 +60 T>C** | **Intron 5** | **-** | **C/C** | **2** | **1** |
|  | **10** | **IVS5 +102 C>T** | **Intron 5** | **-** | **T/T** | **2** | **1** |
|  | **11** | **IVS5 -79 A>G** | **Intron 5** | **-** | **G/G** | **2** | **1** |
|  | **12** | **IVS5 -13 indel T** | **Intron 5** | **-** | **Del/Del** | **2** | **1** |
|  | **13** | **c.684 T>C** | **Exon 6** | **N228N** | **C/C** | **2** | **1** |
|  | **14** | **IVS6 -11 G>A** | **Intron 6** | **-** | **A/A** | **2 (s)** | **2*** |
|  | **15** | **c.822 C>T** | **Exon 7** | **G274G** | **T/-** | **2 (s)** |  |
|  | **16** | **c.891 C>T** | **Exon 7** | **P297P** | **=/T** | **2*** | **2** |
|  | **17** | **IVS7 +28 T>C** | **Intron 7** | **-** | **C/C** | **2** | **3*** |
|  | **18** | **c.1038 C>G** | **Exon 8** | **S346S** | **G/G** | **2*** | **3** |
|  | **19** | **c.1170 C>T** | **Exon 9** | **F390F** | **T/-** | **3*** |  |
|  | **20** | **IVS9 -97 C>A** | **Intron 9** | **-** | **A/-** | **3** |  |
|  | **21** | **IVS11 +72 A>T** | **Intron 11** | **-** | **T/T** | **4*** | **3** |
|  | **22** | **IVS11 +159 A>C** | **Intron 11** | **-** | **C/C** | **4** | **3** |
|  | **23** | **c.1569 T>C** | **Exon 12** | **A523A** | **-/C** |  | **3 (s)** |
|  | **24** | **IVS12 -34 C>T** | **Intron 12** | **-** | **T/T** | **4** | **3** |
|  | **25** | **IVS12 -8 G>A** | **Intron 12** | **-** | **A/-** | **4** |  |
|  | **26** | **c.1680 G>A** | **Exon 13** | **T560T** | **A/A** | **4** | **3** |
|  | **27** | **c.1886 A>T** | **Exon 14** | **Y629F** | **T/T** | **4** | **3** |
|  | **28** | **IVS14 +15C>T** | **Intron 14** | **-** | **T/T** | **5 (s)** | **3** |
|  | **29** | **IVS14 +85 G>A** | **Intron 14** | **-** | **=/A** | **5*** | **3** |
|  | **30** | **IVS14 +90 A>G** | **Intron 14** | **-** | **=/G** | **5** | **3** |
|  | **31** | **IVS14 +128 C>T** | **Intron 14** | **-** | **T/-** | **5*** |  |
|  | **32** | **c.1911C>T** | **Exon 15** | **V637V** | **T/-** | **5 (s)** |  |
|  | **33** | **c.1919 A>G** | **Exon 15** | **E640G** | **G/-** | **5 (s)** |  |
|  | **34** | **c.1932 G>A** | **Exon 15** | **V644V** | **A/A** | **5*** | **3** |
|  | **35** | **c.1969 G>A** | **Exon 15** | **V657I** | **A/A** | **6*** | **3** |
|  | **36** | **c.1974 G>C** | **Exon 15** | **P658P** | **C/-** | **6 (s)** |  |
| ***SDHB*** | **1** | **c.18 A>C** | **Exon 1** | **A6A** | **C/-** | **1*** |  |
|  | **2** | **c.65 G>C** | **Exon 1** | **C22S** | **C/-** | **1 (s)** |  |
|  | **3** | **c.170 A>G** | **Exon 2** | **H57R** | **G/-** | **1 (s)** |  |
| ***SDHC*** | **1** | **IVS1 +9 indel GT** | **Intron 1** | **-** | **Ins/Ins** | **1*** | **1*** |
|  | **2** | **IVS1 -96 C>T** | **Intron 1** | **-** | **T/-** | **1*** |  |
|  | **3** | **IVS2 +186 T>C** | **Intron 2** | **-** | **C/-** | **1** |  |
|  | **4** | **IVS2 +248 G>A** | **Intron 2** | **-** | **A/-** | **1** |  |
|  | **5** | **IVS2 -23 C>G** | **Intron 2** | **-** | **G/-** | **1** |  |
|  | **6** | **c. 354 T>C** | **Exon 5** | **F118F** | **C/-** | **1 (s)** |  |
|  | **7** | **c.633 C>G** | **Exon 6** | **3'-UTR** | **G/G** | **1*** | **1** |
|  | **8** | **c. 757 C>G** | **Exon 6** | **3'-UTR** | **G/-** | **1** |  |
| ***SDHD*** | **1** | **IVS2 -29 A>G** | **Intron 2** | - | **G/-** | **1*** |  |
|  | **2** | **c. 204 C>T** | **Exon 3** | **S68S** | **T/-** | **1** |  |
|  | **3** | **c. 1093 C>T** | **Exon 4** | **3'-UTR** | **T/-** | **1*** |  |
|  | **4** | **c. 1279 T>C** | **Exon 4** | **3'-UTR** | **C/-** | **1 (s)** |  |
|  | **5** | **c. 1283 A>G** | **Exon 4** | **3'-UTR** | **G/-** | **1** |  |

(a) Coding and untranslated region (UTR) variants are numbered relative to the first nucleotide of the translation initiation codon, which is +1.

(b) The sign ‘=’ denotes allelic variants with equal population frequency.

(c) AA=African-American samples, CC=European-American samples

(d) Variants in the same haplotype block (HB) are denoted by identical numbers and the tagging variants are marked by a star (*) sign.

(e) ‘(s)’ denotes variants that were observed only in one chromosome (singleton) in that sample set.
