# Supplementary material for: Sequence variation in human succinate dehydrogenase genes: evidence for long-term balancing selection on SDHA
Source: BMC Biol. 2007 Mar 21;5:12. doi: 10.1186/1741-7007-5-12 (PMC1852088; doi:10.1186/1741-7007-5-12)
Supplement: Additional file 2 — Additional Table 2 – Sequence variants and fixed differences in chimpanzee SDHA [file 1741-7007-5-12-S2.doc]

**Additional Table 2 -Sequence variants and fixed differences in Chimpanzee *SDHA***

| **Sequence type** | **Sequence** | **Location** | **Coding effect** | **Minor chimp allele of variants or the human allele of fixed differences** | **Minor allele frequency** |
| --- | --- | --- | --- | --- | --- |
| **SNP** | **IVS4 +5 A>T** | **Intron 4** | **-** | **T** | **0.14** |
| **SNP** | **IVS4 +10 A>G** | **Intron 4** | **-** | **G** | **0.19** |
| **SNP** | **IVS4 +25 T>C** | **Intron 4** | **-** | **C** | **0.11** |
| **Fixed difference** | **IVS4 +54 G** | **Intron 4** | **-** | **A** | **-** |
| **Fixed difference** | **IVS4 +78 C** | **Intron 4** | **-** | **T** | **-** |
| **Fixed difference** | **IVS4 -118 G** | **Intron 4** | **-** | **A** | **-** |
| **Fixed difference** | **IVS4 -33 A** | **Intron 4** | **-** | **G** | **-** |
| **Indel** | **IVS5 -144 ACAGTG >T** | **Intron 5** | **-** | **ACAGTG** | **0.375** |
| **Fixed difference** | **IVS5 -111 C** | **Intron 5** | **-** | **A** | **-** |
| **Fixed difference** | **IVS5 -80 C** | **Intron 5** | **-** | **T** | **-** |
| **SNP** | **c.708 A>G** | **Exon 6** | **A236A** | **G** | **0.06** |
| **SNP** | **c.1629 T>C** | **Exon 12** | **Y543Y** | **C** | **0.11** |
| **SNP** | **IVS2 -33 G>A** | **Intron 12** | **-** | **A** | **0.08** |
| **Fixed difference** | **c.1752 G** | **Exon 13** | **A584A** | **A** | **-** |
| **SNP (s)** | **IVS13 +3 G>C** | **Intron 13** | **-** | **C** | **0.03** |
| **Fixed difference** | **IVS13 +18 A** | **Intron 13** | **-** | **G** | **-** |
| **SNP*** | **c.1932 A>G** | **Exon 15** | **V644V** | **G** | **0.28** |
| **SNP** | **c.2086 G>T** | **Exon 15** | **3’-UTR** | **T** | **0.28** |

(s) denotes a singleton variant; * denotes a variant shared with humans.
