# Supplementary material for: Sequence variation in human succinate dehydrogenase genes: evidence for long-term balancing selection on SDHA
Source: BMC Biol. 2007 Mar 21;5:12. doi: 10.1186/1741-7007-5-12 (PMC1852088; doi:10.1186/1741-7007-5-12)
Supplement: Additional file 3 — Additional Table 3 – Locus-by-locus HKA tests of SDHA versus neutrally evolving loci [file 1741-7007-5-12-S3.doc]

**Additional Table 3 -Locus-by-locus HKA tests of *SDHA* versus neutrally evolving loci**

| Locus | Reference | bp compared | Segregating sites in human | No. of samples | bp different between chimp-human | HKA prob. * |
| --- | --- | --- | --- | --- | --- | --- |
| *SDHA* | This study | 2832 | 19 (silent or non-coding) | 45 | 8 | - |
| Noncoding region at 1q24 | [30] | 8991 | 48 | 61 | 56 | 0.128 |
| Beta-globin promoter | [33] | 6076 | 41 | 18 | 78 | 0.067 |
| Noncoding region at 22q11 | [31] | 9091 | 75 | 64 | 134 | 0.010 |
| Noncoding region at Xq13.3 | [32] | 10200 | 33 | 69 | 94 | 0.014 |

*P* values are calculated by direct HKA mode in DNAsp software. Non-coding or silent variants (n=33) in the full gene sequence (5255 bp) were used for intra-specific variation in *SDHA*.
