# Supplementary material for: Sequence variation in human succinate dehydrogenase genes: evidence for long-term balancing selection on SDHA
Source: BMC Biol. 2007 Mar 21;5:12. doi: 10.1186/1741-7007-5-12 (PMC1852088; doi:10.1186/1741-7007-5-12)
Supplement: Additional file 5 — Additional Table 5 – PCR oligonucleotide primers and the amplicon sizes for SDHA, SDHB, SDHC, and SDHD exons [file 1741-7007-5-12-S5.doc]

**Additional Table 5- PCR oligonucleotide primers and the amplicon sizes for *SDHA*, *SDHB*, *SDHC* and *SDHD* exons**

| **Gene-Exon** | **Forward (5’-to-3’)** | **Reverse (5’-to-3’)** | **Size** | **No. of Fixed nucleotide differences between *SDHA* and its duplicated pseudogenes within amplicon (excluding primer binding sites)** |
| --- | --- | --- | --- | --- |
| *SDHA*-1 | CGCTCGACTCCGGCGTGGT | TCCCGATCCCGGGACAGA | 314 | 23,35,41,48 |
| 2 | GTTTGCAAGGGGAAATTACTATC | TTTACTTGATCTAAATTTAAATCTAG | 420 | 6,7,10 |
| 3 | AGGTGCGAATGTGCCCACC | TAAGGCACAGCAAGAGGCCAT | 466 | 11,12,14 |
| 4 | GCCTGGAAGACAAAGTTGGC | TTCTTTTGGCTGCCACATCC | 308 | 12,14,15 |
| 5 | TCTTTTTCCGTTATGAACTATGC | CGCTGCATGCGCCCCACGCT | 498 | 31,36,37 |
| 6 | CTGCCTTGTCTGTACTGCTC | CAAGGCCTGTGTCTAAACCGT | 460 | 15,16,18 |
| 7 | TGTCCATTCTGTGATCTCACC | AGTTAGGACCTGAGAGCTGA | 344 | 29,32 |
| 8 | TCTAGCAATTGTTAGGTAATAAAT | AGACGCCTGCCAGCAGCTA | 351 | 10,11 |
| 9 | AGGTCTCCTGCCGTTGCCG | TGCGAGGTGGGCCCCGTC | 315 | 11,11 |
| 10 | GTAGAGATGGGGTTTCACCG | AAAAAGAAAAAAGACTTTCTAACG | 463 | 12,36 |
| 11 | GTTAGCTCAGGAGACTTACAG | CACTTGGAAGTGGAAGCTGAAT | 540 | 20,34,41 |
| 12+13 | TCCTAATTTTTCTGTTTAATTTATG | CTGAGTCCCACCAGGTGGC | 666 | 21,34,34 |
| 14 | GCTCTGTTAGAGTAATAAGAAAC | GAAGCCCGACTGCTGTCG | 327 | 4,9,9 |
| 15 | GAATCTTAAAGTTCACATGCCA | GGCAAGCTCCCAGCCACTA | 363 | 7,12,13 |
| *SDHB*-1 | GGTCCTCAGTGGATGTAGGC | CTTGCCCTATGCTTCCTCAG | 269 | - |
| 2 | CCAGCAAAATGGAATTATCTTGT | TCTCCTTCAATAGCTGGCTT | 232 | - |
| 3 | GAACGTTACATAAATACCACTGGA | CTATCAGCTTTGGCCAGC | 201 | - |
| 4 | CAGCAAGGAGGATCCAGAAG | ACAAATCCTGCCCTGAAAAA | 315 | - |
| 5 | CAGTGTCCAAGAAATGGGGT | TGCCAGTTCCTCTCCAGAAT | 335 | - |
| 6 | CCTCTCTTTTCTCCCCATAC | CAGCAATCTATTGTCCTCTT | 200 | - |
| 7 | AGCTAATCATCCCTGGTTTT | TTGTGAGCACATGCTACTTC | 215 | - |
| 8 | GTGGGTTTTCCCTTTCAGTT | CGGCAAGTAAAGGAACAGGT | 320 | - |
| *SDHC*-1 | CACATGACACCCCCAACCC | CTGCCCAGGCACAGGATAAACA | 216 | - |
| 2 | GTGTTTGATTAACTCTATTTTGCAT | CTATTGCTCTTCCCTAAGGAA | 645 | - |
| 3 | ACGTTATGCAAAATATTAAACCAAGT | TCTAGATTCTCTGGCTCCA | 201 | - |
| 4 | GTTTATATTTTTGCCAAGATAGACTC | CCAAGTTTTTCAAAGAAGCACA | 195 | - |
| 5 | TCATATTAGTTGTAACTTATGAGCAGC | CTCCCCACTCCCTTCACAG | 269 | - |
| 6 | GCGCTTTTCTCTAGAATCATG | CCCAGGGCAGAAGCCACAGAGCT | 602 | - |
| *SDHD*-1 | TCGTCGTGGGTGGGAA | CAGTGGTCATTGCTGTGATT | 255 | - |
| 2 | GTTATCCCCTATTTATTGTTAA | TCTGCCCAAAGGTGTAAACTA | 373 | - |
| 3 | GAGATAGCTTCTCTCAACTAC | GGGCATTTCAATCAACTTCTC | 409 | - |
| 4A | AAGGCTATACAGAATCCCCT | AGAGTATTTCCCTTTGTCCA | 725 | - |
| 4B | GGTTGCCTCCCAGCTTCTT | CATGCTGTGGATGC | 659 | - |
